# Psychological Safety in High-Performance Sport: Contextually Applicable?

**DOI:** 10.3389/fspor.2022.823488

**Published:** 2022-05-09

**Authors:** Jamie Taylor, Dave Collins, Michael Ashford

**Affiliations:** ^1^Grey Matters Performance Limited, Stratford upon Avon, United Kingdom; ^2^School of Health and Human Performance, Faculty of Science and Health, Dublin City University, Dublin, Ireland; ^3^Moray House School of Education and Sport, The University of Edinburgh, Edinburgh, United Kingdom; ^4^Faculty of Health and Life Sciences, Coventry University, Coventry, United Kingdom

**Keywords:** criticality, pragmatism, sport psychology, elite sport, talent development, high performance milieu

## Abstract

In recent years, high-performance sport has seen a rising interest in Psychological Safety, a construct with a strong empirical basis in certain business contexts. As research and practice interest grows in PS, there are early indications of practitioners and, to a lesser extent research, treating the construct as being universally transferable. We offer three central concerns with this situation. Firstly, it seems that a variety of different interpretations in use may limit the practical application of the construct. Secondly, a concern that not all dimensions of PS are transferable or applicable in the HPSs context, especially for athletes. Finally, emerging evidence from outside of sport suggests potential downsides to the perceptions of PS in a performance/selection sets. We suggest that, as with all theories and constructs, there is a pressing need for nuance and context-specific evidence in how researchers and practitioners approach transferability plus, perhaps, a little more understanding of the real-world high-performance context.

## Introduction

High-performance sports (HPSs) have a dubious history with the representation of nuanced constructs. Over time, it has seen a variety of fashionable concepts presented as panaceas (e.g., growth mindset, grit, and 10,000 h). These concepts sometimes have empirical support (Duckworth et al., 2007; Yeager and Dweck, 2012), but often oversimplified, whilst others do not (e.g., MBTI personality profiling and learning styles—Bailey et al., 2018). Importantly, however, whilst some of the constructs in the former category have their place as part of a broader whole, this nuance is often missed by practitioners looking for an edge to develop performance or perhaps in the literature (Burgoyne et al., 2020). In addition, with social media becoming the information source of choice for many practitioners (MacNamara and Collins, 2015), the risks of hyperbole beyond reality grow stronger (Stoszkowski and Collins, 2016) with the original meaning and mechanism of the construct getting lost, often in a plethora of promotional presentation. As a result, eager practitioners frequently pick-up and apply constructs without a sound underpinning of declarative knowledge sufficient to make decisions about why or why not, when or when not, they might be appropriate for that particular context (Collins et al., 2012, 2015).

In recent times, psychological safety (hereafter PS) has recently emerged as a ‘hot topic' in the HPS milieu, becoming common jargon in sporting organizations. Notably, however, there has been little empirical research into the concept in talent development or elite sporting contexts (Smittick et al., 2019). A quick Google search will return a variety of examples and materials that highlight this issue, often with the construct has been presented as an essential feature of the HPS milieu (Leaders Virtual Roundtables: Psychological Safety, 2017; Roberts and Paquette, 2021). Even in published research, PS appears to be implicitly desirable (e.g., as shown in Ref. Henriksen, 2015 and Morgan et al., 2019). In essence, and as with some of the earlier ideas, there is a danger of the construct being underexplored and misapplied. For instance, in a time where all of HP sport is more aware of athlete welfare concerns, if athletes are told that PS is an essential feature of their experience, how does the coach manage selection or essential challenge? Additionally, how does the athlete respond when they are judged for their performance? And, if a lack of PS is a performance enhancer, how is this approached?

We, therefore, suggest the current situation presents three significant concerns. Firstly, the variety of different definitions detract from the meaning of the construct and therefore limits its practical application. Secondly, insufficient critical consideration of transferability to HPS settings, especially in a performance/selection sets. Thirdly, to this point, a lack of awareness, or consideration of the potential disadvantages of PS.

From the outset, it is important to state that this review in no way seeks to invalidate the well-established empirical base in organizational psychology, especially in the realm of “knowledge work” amongst teams of between 5 and 20 people (Edmondson, 2004). For an in-depth review of the literature in this context, the reader is directed toward a number of meta-analyses (Frazier et al., 2017) and reviews (Edmondson and Lei, 2014; Newman et al., 2017). Rather, our focus in this critical review is to question the *universal* adoption of PS in HPS contexts without a critique of *transferability* or *applicability* within such environments.

## Conceptual Clarity

Although initially conceptualized as an individual construct, the most prominent definition of PS is the group-level approach of Edmondson (1999, p. 350): “a shared belief by members of a team that the team is safe for interpersonal risk taking.” This definition is underpinned by seven scale items (with R representing items that are negatively scored):

- If you make a mistake on this team, it is often held against you (R)
- Members of this team can bring up problems and tough issues
- People on this team sometimes reject others for being different (R)
- It is safe to take a risk on this team
- It is difficult to ask other members of this team for help (R)
- No one on this team would deliberately act in a way that undermines my efforts
- Working with members of this team, our unique skills and talents are valued and utilized (Edmondson, 1999, p. 382).

It is this definition and the associated factor scale that has guided research in sport so far (Smittick et al., 2019; Fransen et al., 2020; Gosai et al., 2021). Building on this and seemingly capturing a dual effect, the most recent Edmondson definition states:

“Psychological safety describes a belief that neither the formal nor informal consequences of interpersonal risks, like asking for help or admitting a failure, will be punitive. In psychologically safe environments, people believe that if they make a mistake or ask for help, others will not react badly” (Edmondson, 2018, p. 15).

In essence, whilst being a multi-dimensional construct, there appear to be two resulting shared group perceptions. Firstly, people will not be rejected for being themselves, asking for help, or saying what they think. Secondly, mistakes will not lead to negative consequences for the individuals involved, which consequently, make them feel safe to experiment (Edmondson, 1999). Thus, PS is an emergent *social* construct arising from multiple interpersonal interactions over time, making it an unstable feature of shared perception, both dynamic and fragile, and reliant on a variety of individual, group, and contextual factors (Edmondson, 2004; Kolbe et al., 2020).

Notably, and highlighting that we might not all be saying the same thing, other definitions used in the sports literature, including McLaren and Spink's (McLaren and Spink, 2022, p. 6) definition that used “indicators of PS”: “member's beliefs regarding support and flexibility from leaders, clarity with respect to one's role in the group, and the opportunity for self-expression.” Similarly, a multi-national position statement on athlete mental health in HPS suggested the need to “improve the PS of pre-, during-, and post- games high performance environments” (Henriksen et al., 2020, p. 16). Whilst we support the increasing focus on athlete mental health, based on the research base currently available, we are aware of very little evidence that increased PS mitigates risk factors for mental health illness (e.g., Ulusoy et al., 2016). Different again was the definition of Bean et al. (2020) who offered a definition of physical and psychological safety: “an environment that allows youth to feel both free from being physically harmed and accepted and respected” (Bean et al., 2020, p. 40). Indeed, this lack of conceptual/consistent clarity was noted in a recent systematic review across the sporting literature, suggesting that there was a need for the construct to be defined specifically in sport (Vella et al., 2022). Their review suggested an alternative definition suggesting “psychological safety in sport is the perception that one is protected from, or unlikely to be at risk of, psychological harm in sport” (Vella et al., 2022, p. 15). Psychological harm in this case was inclusive of fear, threat, and notably, in contrast to the organisational literature was changed to an individual level construct.

Finally, and again highlighting the conceptual challenges, in the unpublished Google report “Project Aristotle,” PS was found to be “by far and away from the most important dynamic” correlated with team performance (Rozovsky, 2015). PS was again defined subtly differently, emphasizing voice behavior in response to errors, rather than the poor performance itself: “a belief that a team is safe for risk-taking in the face of being seen as ignorant, incompetent, negative, or disruptive. In a team with high PS, teammates feel safe to take risks around their team members” (Rozovsky, 2015). Although some may read this point as splitting hairs, in the HPS setting, the difference in relation to performance is fundamental.

Of course, multiple definitions of the same term are hardly a new problem in psychology, (e.g., creativity—Cropley, 2011) and subsequent lay (mis)interpretation is a barrier to effective use (Lucas and Nordgren, 2021). The conceptual journey of mental toughness is a telling example in sport (Gucciardi, 2017). Notably, Edmondson and Lei (2014) raised concerns about the use of measures that were inconsistent with the 1999 definition. We, therefore, need to strive for conceptual clarity, especially as the construct appears to be both dynamic and fragile. In lieu of this clarity, there is a risk of conceptual leakage and the robust work of Edmondson and colleagues from alternate sectors losing meaning.

## Is it Transferable? Confounding Contextual Factors

Our second concern is the extent to which the entirety of the construct can be transferred across contexts. In our role as practitioner-researchers, we adopt a pragmatic approach where researchers are encouraged to design methods that consider transferability across contexts (Cruickshank and Collins, 2017; Jenkins, 2017). Transferability challenges the positivist assumption that findings offer widespread generalizability and instead, emphasizes the critical consideration of applicability across contexts. For this reason, we suggest a need to consider the complexity inherent in the HPS milieu and the stark differences to other work environments. A primary concern is the extent to which perceptions of safety, including the lack of fear of judgement (Edmondson, 1999) are possible in HPS (cf. Taylor and Collins, 2021a). As has been suggested: “elite sport is inherently unsafe” (Portch, 2021). Thus, it is far from clear whether *all* dimensions of PS are contextually realistic or indeed desirable. Therefore, taking the earlier mentioned scale items of Edmondson (1999), there appear multiple challenges. For the athlete, there is no getting away from the real-world consequences that can occur following underperformance (loss of funding, sponsorship, etc.). As such, there comes a point where taking risks and making mistakes *cannot* be “safe” (Bstieler and Hemmert, 2010).

In addition, we need to be realistic and accept that the talent development journey toward HPS is subject to the same pressures. Selection is a reality of the domain, just as it is in education, dance, or indeed any job interview. Additionally, as increasing scrutiny is applied to sports to formally justify selection criteria, there is pressure for the use of ‘objective' measures that can be later defended (Johnston et al., 2021). As such, if performance data are used in this way, mistakes will inevitably be held against people. Whilst we do not completely recommend this type of approach, we would suggest that HPS takes account of 20 years of research and consider a range of factors that are less “measurable” (Abbott et al., 2005; Till and Baker, 2020). Notably, these may sometimes be informed by an individual's cultural fit. Although hardly revolutionary, this type of approach also became fashionable when several highly successful sports teams had their success (perhaps spuriously!) attributed to a so-called “FIFO”^1^ approach. A high-profile example being the New Zealand All Blacks and their often cited “no dickheads” policy (Kerr, 2013). In the real world, whilst it is clear that some level of difference is perfectly acceptable and indeed desirable, how much difference is too much is socially constructed (Hu et al., 2021). Either way, in the selection or development context, it is difficult to see how PS can be universally applicable.

### Cultural Mediators

In addition, the extent to which an environment is individualistic also appears to be a moderator for the performance-enhancing impact of PS (cf. Edmondson, 2008). This creates another challenging dimension for widespread transferability; elite sport is of course a broad church with a variety of different national cultures, sporting cultures, and group subcultures (Hughson, 2009). If as identified, ‘super elite' athletes *are* selfish and ruthless (Hardy et al., 2017), this is antithetical to a collectivist environment where the organization's goals are superordinate. Under these conditions, a “high PS climate coupled with a strong individualistic group culture may even create a “zone of egocentrism” where the motivation for group success is reduced” (Deng et al., 2017, p. 1137). This also poses a challenge for the talent development context, where an established marker of high-quality practice is a focus on the individual, rather than collective performance (Martindale et al., 2007; Henriksen, 2010).

There is also a need to consider the multiple sub-groups that populate the various levels of HP teams and organizations. Most PS research focuses on intact teams, with an overarching climate and limited individual differences within a group. Studies have also tended to aggregate data across team members, without concern for the social complexity of various sub-groups (Roussin et al., 2016). In contrast, within the HPS setting and its additional complexity of various hierarchies of performers, coaches, and staff, there is the potential for multiple sub-groups to exist (those who are selected, or in receipt of higher levels of funding, or salary). On this basis, along with highly variable individual responses to events, it is problematic to classify an environment based on the shared perceptions of all those who inhabit it (Taylor and Collins, 2021b). Thus, any call to create psychologically safe environments appears overly simplistic (Deng et al., 2017). The point is that the application of PS seems to *depend* on a wide variety of different contextual factors.

## Potential Downsides—Is PS Universally Positive

One key issue across varied interpretations relates to the levels of challenge. For example, Edmondson (2008) proposed a 2 × 2 matrix, suggesting that low levels of accountability could *mediate* PS, creating feelings of ‘comfort', where people “really enjoy working with each other, but do not feel challenged. Nor do they work very hard” (Edmondson, 2008, p. 6). Furthermore, Edmondson and colleagues suggested a number of other potential contextual boundary conditions, such as size or complexity of the team, level of certainty regarding the nature of the work, the need for task interdependence, and fluidity of the group (Edmondson, 2004). Speculating on the downsides of the construct: “it is also possible that the effects of PS become less pronounced over time as people become too comfortable with each other” (Edmondson and Lei, 2014, p. 39). Extending this concern, only a limited number of empirical studies have considered the boundary conditions of PS (Newman et al., 2017), as stated by Frazier et al. (2017, p. 532): “there has been an inordinate focus in prior empirical work on examining the positive outcomes of psychological safety.”

Notably, in the limited number of studies that *have* considered these conditions, PS appears far from universally positive. For example, in a large-scale longitudinal study of 170,000 teachers, Higgins et al. (2020) found PS to be not necessarily supportive of organizational performance over time. Rather, it appeared that optimal conditions for performance identified relatively low PS against high perceptions of accountability. Similarly, Deng et al. (2017) found evidence that high levels of PS *negatively* impact motivation and effort levels. There has even been the suggestion that teams high in PS were more likely to engage in unethical behavior (Pearsall and Ellis, 2011). This body of work offers further evidence that PS is fragile and, depending on the context, has the potential to hinder performance. Indeed, for optimal long-term development in HPS, there is a wide body of evidence that supports the potentially beneficial exposure to environments where it *is not* entirely safe to fail (Collins et al., 2016; John et al., 2019; Taylor and Collins, 2021b).

Yet, without wishing to throw the baby out with the bathwater, there are some contexts where perceptions of PS may be a useful feature of progress. Highlighting the complexity of the issue, there is a clear contrast between the experience of an athlete at a major international competition, or approaching a funding/contract decision, and an athlete-coach pairing experimenting with a technical adjustment 12 months into a quadrennial cycle. In the latter, perceptions of PS may be essential in allowing for “safe” risk-taking and low performance to not be held against the athlete (or coach). Similarly, we might want a situation where a new signing to a team is made to feel welcome by peers and accepted despite different non-performance-relevant characteristics. There is also evidence from NCAA level basketball of associations between coach civility, perceptions of psychological safety, and team performance (Smittick et al., 2019). Thus, it seems clear that PS cannot apply *universally* throughout a season, quadrennial, or indeed talent development pathway.

## Voice vs. Performance Consequences

Therefore, despite the contextual challenges, we would also suggest that there are dimensions of PS that warrant further investigation. The dual effect suggested by Edmondson (2018) of voice and performance consequences presents a useful framing. It would seem very appropriate that a high-performing HPS environment would be one where people regularly raise tough issues or offer feedback for performance enhancement (Din et al., 2015). This idea runs parallel to the concept of the Zone of Uncomfortable Debate, which suggests a group responsibility for honest discussion (Bowman, 1998). The point of contention is the extent to which PS scale factors (Edmondson, 1999) need to be prevalent in an HP environment to encourage robust discussion. Deductively, it would seem appropriate for problems to be reported and mistakes addressed, even if they bring people's competence into question (Edmondson, 2018). Yet, given that the difference between disruptive and productive disagreement seems to be socially constructed (Edmondson and Besieux, 2021), we need more than correlational research to inform practice. It may be the case that PS is more appropriate for roles in HPS where the context of work *is* closer to that of the knowledge worker (e.g., coaching/interdisciplinary teams).

## Moving Forward

Far from disparaging the long and significant body of research that underpins PS, we suggest that, as *should* be the case with all constructs, it is important to consider the appropriateness of use (cf. PJDM—Martindale and Collins, 2013). This is especially so as the correlates of PS are complex and not always wholly positive (Taylor and Collins, 2020). As such, we repeat a recent call for an increased level of criticality, especially for those seeking to develop evidence-informed practice (Stoszkowski et al., 2020).

Our main point is that as a global construct, PS cannot be considered universally applicable or positive in HPS. Thus, for the researcher, it may be the case that the construct is redefined for applicability and the emphasis on performance consequence removed, or it may be more relevant for certain roles or contexts. We would suggest that this would be convoluted means of retrofitting the construct. We would suggest that this would be convoluted and undermine a well-established construct. Rather than devoting energy to changing a well-established definition and further muddying the waters, we should instead consider the mechanisms underpinning the desirable outcomes of voice behavior and performance improvement. In both cases, there are fewer global constructs that offer significant transferability. For example, implicit voice theory (Detert and Edmondson, 2011), role clarity (e.g., Bray and Brawley, 2002), and positive conflict (e.g., O'Neill et al., 2013). This is especially relevant when, in a context of increasing concerns about mental health, embracing such athlete-friendly constructs is highly desirable, both socially and professionally.

More broadly, given the recent surge in interest in PS, we urge caution to those that might see universal applicability, especially using constructs from outside of HP sport without considering boundary conditions (Edmondson and Lei, 2014). Much like other concepts (e.g., athlete-centredness—Alder, 2018), there is a risk that utility is neutralized, making them a little more than buzzwords. The key for us, and other researchers, is an understanding and focus on the mechanisms that underpin the phenomena (Deng et al., 2017; Collins et al., 2019).

## Author Contributions

All authors made substantial contributions to the conception and design of the manuscript, including drafting and revising it. All have approved this version for publication and agree to be accountable for all aspects of the work.

## Funding

Funding for open access publication was granted through Dublin City University.

## Conflict of Interest

JT was employed by Grey Matters Performance Limited. The remaining authors declare that the research was conducted in the absence of any commercial or financial relationships that could be construed as a potential conflict of interest.

## Publisher's Note

All claims expressed in this article are solely those of the authors and do not necessarily represent those of their affiliated organizations, or those of the publisher, the editors and the reviewers. Any product that may be evaluated in this article, or claim that may be made by its manufacturer, is not guaranteed or endorsed by the publisher.

## Footnotes

^1^So called: “Fit in or fuck off”.
